# Anticoagulation management during low flow states in pediatric bivalirudin extracorporeal membrane oxygenation

**DOI:** 10.3389/fped.2026.1902882

**Published:** 2026-08-11

**Authors:** Ahmad T. Hamed, Miranda Edmunds, Matthew Douds, Michael Lahart, Ahmed S. Said

**Affiliations:** 1 University of Mississippi Medical Center, Jackson, MS, United States; 2 Division of Pediatric Critical Care Medicine, Washington University in St. Louis, St. Louis, MO, United States; 3 Mechanical Assist Department, St. Louis Children’s Hospital, St. Louis, MO, United States, United States; 4 Department of Pharmacy, St. Louis Children’s Hospital, St. Louis, MO, United States

**Keywords:** bivalirudin, clamp trial, direct thrombin inhibitor, ECLS, extracorporeal membrane oxygenation, heparin, low flow

## Abstract

**Introduction:**

There is growing utilization of direct thrombin inhibitors (Bivalirudin) for primary pediatric extracorporeal membrane oxygenation (ECMO) anticoagulation. During low-flow states, proteolytic inactivation of bivalirudin decreases its efficacy. We describe our experience with unfractionated heparin (UFH) boluses, prior to and during low-flow and clamp trials, with bivalirudin anticoagulation.

**Methods:**

Retrospective chart review of all pediatric ECMO runs from Jan 2020 till May 2021 anticoagulated with bivalirudin, that underwent low-flow or clamp trials. ECMO flows, UFH boluses, bivalirudin infusion rates, anticoagulation profiles, hemoglobin values and blood product administration were recorded before and after trials, as well as circuit interventions and stroke incidence.

**Results:**

Thirty-one ECMO runs met inclusion criteria, with 65 low-flow or clamp trials. Median UFH bolus dose was 25 units/kg, inter-quartile range (IQR) of [15, 50]. Median blood product volumes 24 h pre- and post-trial were 15 mL/kg [IQR 13, 20] vs. 15 mL/kg [IQR 10, 20]. Eight post-trial circuit interventions were performed (6 connectors, 2 pump exchanges) in 4 patients, and 3 ischemic strokes occurred in 3 separate patients. There were no significant differences before and after trials in coagulation profiles, bivalirudin infusion rates, or hemoglobin values.

**Conclusion:**

UFH boluses during low-flow states in pediatric ECMO with bivalirudin anticoagulation are safe, without increased bleeding, circuit deposit burden requiring circuit interventions, or stroke. Prospective multicenter studies are needed to compare this approach to other anticoagulation strategies during low-flow and clamp trials, including UFH alone and bivalirudin without adjunctive UFH, and to further define their optimal dosing and safety profiles.

## 1 Introduction

Despite the growing utilization of extracorporeal membrane oxygenation (ECMO), there continues to be great variability between centers in their approach towards circuit anticoagulation (1). Unfractionated heparin (UFH) has historically been the commonly used agent, despite its known risk factors (1, 2). These include its age-dependent reliance on antithrombin in young children, inability to break down bound thrombin, and heparin induced thrombocytopenia (HIT), all of which have driven the interest in alternatives such as direct thrombin inhibitors (DTIs) (3). Widespread adoption of this alternative has been slow, in part due to the lack of experience using DTIs compared to UFH (4). Limitation in the consistency of current anticoagulation laboratory testing makes it difficult to draw predictive statistical comparisons between both agents (5, 6). In addition, the anticoagulant response to UFH can be highly variable because of interpatient differences in pharmacokinetics and antithrombin availability, making consistent therapeutic dosing challenging during ECMO (7). Some of the benefits of DTIs like bivalirudin, is its ability to inhibit both free and bound thrombin without the need for a cofactor, a lower immunogenic potential, and a non-inferior anticoagulant effect compared to UFH, even with longer ECMO runs (4, 6). A downside of bivalirudin is the unavailability of a reversal agent, however, it has a relatively shot half-life of 25 min. Around 20% is renally excreted, while the remainder is cleared by enzymatic proteolysis, which may help in a more predictable dose response in patients with reduced creatinine clearance (8). This plasma-based enzymatic proteolysis is thought to be enhanced during states of low flow or stagnation. Additionally, thrombin itself is capable of cleaving bound bivalirudin, further decreasing its efficacy in such conditions (9).

The clinical relevance of bivalirudin pharmacokinetics is of higher significance in pediatric ECMO, where circuit flows are lower than those in adults. This is more pronounced during weaning trials (ex: low-flow, clamp) that are used to assess readiness for decannulation. These could result in frequent circuit component changes, bleeding, increased blood product exposure, and interruptions in support, all due to a paradoxically increased risk of clot deposition (10). To account for this, our institutional practice is to administer heparin boluses immediately prior to a weaning trial, while the bivalirudin infusion continues. However, balancing the risk of significant patient bleeding when exposed to both anticoagulants, with that of circuit clot deposition, is not well described. In this study, we review our experience using heparin boluses with bivalirudin anticoagulation during low flow states, with the primary aim of describing the incidence of associated patient bleeding, circuit clot deposition and patient thrombosis. Our secondary aim is to potentially identify a safe heparin bolus dose associated with lower circuit thrombus formation, while minimizing risk for patient bleeding.

## 2 Methods

### 2.1 Study population

We conducted a retrospective chart review of all ECMO patients in the pediatric (PICU) and cardiac intensive care units (CICU), from January 2020 to May 2021. Patients included were those on bivalirudin as the primary anticoagulant, ages 1 day to 18 years, on veno-arterial (VA) ECMO, who underwent low-flow and/or clamp trials during their ECMO run. Institutional Review Board (IRB) approval was obtained, with waiver of informed consent (IRB number 201901020, approval date 1/10/2019).

### 2.2 Materials

All patients in the study were supported on centrifugal pump (CP) based circuits; the majority were either 1/4-inch or 3/8-inch custom Medtronic (Minneapolis, MN) circuits driven by Medtronic Affinity CP cones, all incorporating Cortiva coated circuit tubing. Circuits were selected based on patient size, with <15 kg utilizing 1/4-inch tubing and the Pediatric Quadrox-iD oxygenator (Maquet, Rastatt, Germany), and ≥15 kg utilizing 3/8-inch tubing and the Adult Quadrox-iD (Maquet, Rastatt, Germany) oxygenator. Also included in the study were the Bioline coated Cardiohelp HLS set circuits by Maquet attached to a custom Cortiva coated Medtronic tubing pack. All circuit designs incorporate a 1/4-inch bridge and Terumo (Ann Arbor, MI) CDI System 500 or 550, with an arterial shunt sensor and venous H/S cuvette. Both the Bio-Medicus and Cortiva coated Elongated One-Piece Aortic Cannula (EOPA) Medtronic cannulas were utilized.

### 2.3 Flow and trial definitions

Flow goals for full VA ECMO support were initially set to achieve a cardiac index of 2.5–3.0 mL/min/m^2^, or as clinically applicable. Readiness to separate from mechanical support was assessed by performing low-flow trials, which when successful, were followed by clamp trials, then decannulation. For patients <15 kg on a small ¼ “circuit, low flow was defined as 200 mL/min, while for those ≥15 kg on a larger 3/8” circuit, the target was 500 mL/min. These thresholds represent our institutional ECMO protocol and were established by our multidisciplinary ECMO team as the minimum circuit flow rates considered necessary to maintain circuit patency while allowing assessment of native cardiac function during weaning. Because circuit design, oxygenator characteristics, and institutional practices vary considerably, there are currently no universally accepted definitions of low-flow support. During low-flow trials, to reduce support provided to the patient while maintaining circuit flow, an adjustable J-clamp on the arterio-venous bridge was opened, in addition to controlling pump speed accordingly. Detailed patient hemodynamic variables during the trials were not collected as part of this retrospective review and were therefore not included in the analysis. During clamp trials, this bridge was similarly opened to allow for full recirculation, while the tubing proximal to both cannulae were clamped. Our standard ECMO heparin cannulation dose is 100 units/kg. To reduce the risk of circuit thrombosis during trials, we have selected half that standard heparin dose (50 units/kg), which was administered 10 min prior to trial initiation. For patients deemed a high bleeding risk per the clinical team's assessment, a reduced dose of 25 units/kg was given. The same heparin bolus is repeated for trials lasting longer than 30 min, and at every 30 min interval, so long as no clinically significant bleeding is observed. During the study period, all planned low-flow and clamp trials successfully achieved the target flow rates and were completed without premature termination due to patient or circuit instability. Specifically, no trials were aborted because of inability to achieve target low flow, excessive negative inlet pressures, suction/chattering events, or circuit instability. Trial duration was determined by the treating clinical team based on the patient's clinical response and readiness for decannulation.

### 2.4 Transfusion and anticoagulation definitions

During the study period, bivalirudin was the institutional first-line anticoagulant for all pediatric ECMO patients regardless of ECMO modality or patient age and was not reserved for specific indications such as heparin resistance, antithrombin deficiency, or heparin-induced thrombocytopenia. We utilized our institutional tier-based hemostasis and anticoagulation guideline, which stratifies transfusion and anticoagulation goals per each patients' perceived risk of bleeding or pro-thrombotic state (Supplementary Digital Content S1) (11). In preparation for cannulation, patients received heparin boluses of 50–100 units/kg based on the guideline tier they were assigned. Bivalirudin infusions were typically initiated within one hour of ECMO support, and with starting doses of 0.15–0.2 mg/kg/hr. For patients with renal dysfunction (creatinine clearance <30 mL/min/1.73m^2^), lower starting doses of 0.05–0.1 mg/kg/hr were used, with more frequent laboratory monitoring.

### 2.5 Data collection & outcome measurement

We collected ECMO data including flow rates, frequency and duration of both full support and low-flow trials. We indicated how many trials were immediately followed by decannulation. Anticoagulation data included bivalirudin infusion rates in the 24 h before and after trials, the dose and frequency of UFH boluses administered, and laboratory coagulation parameters such as activated partial thromboplastin time (aPTT) and thromboelastography (TEG) in both time periods. All patients underwent standardized daily TEG monitoring as part of our institutional anticoagulation protocol (Supplementary Digital Content S1). To achieve the study's primary aim, we recorded patient hemoglobin as well as the volume of blood products administered 24 h before and after each trial as a surrogate bleeding parameter. Circuit performance was routinely assessed using inlet pressures, oxygenator transmembrane pressures, post-oxygenator blood gases, and serial visual inspection of thrombus and fibrin deposition. Circuit or circuit component exchange was performed based on the multidisciplinary ECMO team's overall assessment of circuit performance, as no standardized objective thresholds exist to guide intervention. Circuit thrombosis was recorded as any circuit intervention requiring either single-component replacement or complete circuit exchange. Patient thromboembolic events resulting in stroke were recorded when identified on clinically indicated neuroimaging. Routine post-trial neuroimaging was not performed.

### 2.6 Statistical analysis

Continuous variables are presented as median and interquartile range [IQR], and categorical variables are presented as frequency and percentage, unless otherwise specified. Data was not normally distributed; non-parametric tests were used for statistical comparisons. Fisher's exact test was used for non-paired categorical variables. Mann–Whitney *U*-test was used for non-paired analyses of independent categorical variables. Wilcoxon signed-rank test was used for paired analyses, specifically those that account for repeated measurements between ECMO trials. Simple linear regression was used to determine correlation between continuous variables, and coefficient of determination R^2^ was reported. All data analysis was performed using GraphPad Prism (version 10.2.1 for Windows, GraphPad Software, Boston, Massachusetts USA, https://www.graphpad.com). A two-tailed *p*-value less than 0.05 was considered significant.

## 3 Results

### 3.1 Patients and ECMO characteristics

**Figure 1:**
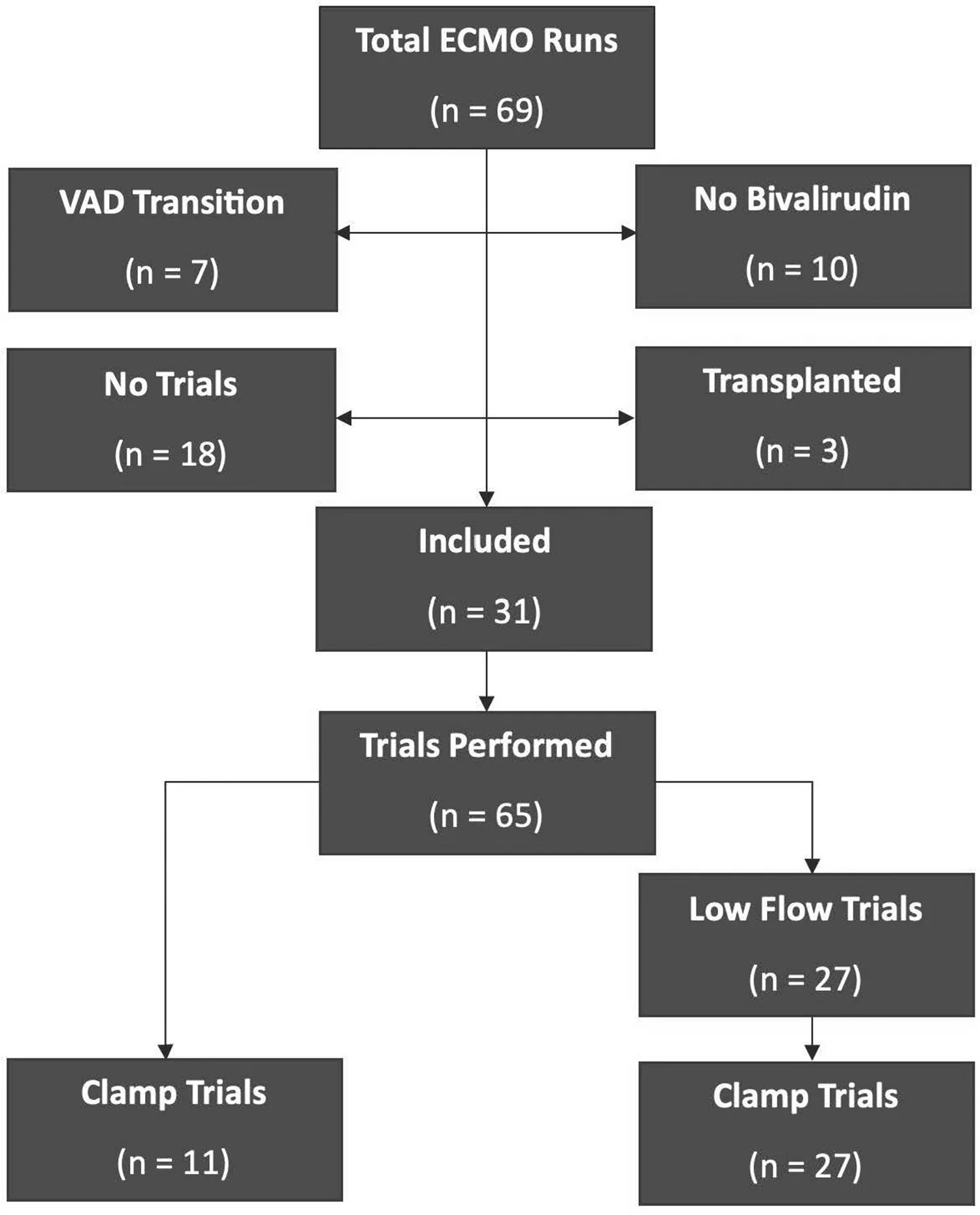
Patient and ECMO run selection flowchart. We excluded patients who were not anticoagulated with bivalirudin as their primary anticoagulant, those who transitioned from ECMO (Extracorporeal Membrane Oxygenation) to VAD (Ventricular Assist Device) support, those who were bridged with ECMO to transplant and those who did not undergo low flow or clamp trials. A total of 31 patients were included with 27 low flow trials followed by clamp trials and 11 additional clamp trials. ECMO, extracorporeal membrane oxygenation; VAD, ventricular assist device.

During the data collection period, a total of 69 ECMO runs were performed. Of these, 31 runs in 30 patients met our inclusion criteria (Figure 1). Reasons for patients not undergoing a trial included being on veno-venous (VV) ECMO, transitioning to a ventricular assist device (VAD), bridging to transplant, no trials performed, or deceased while on support. Patient and ECMO support characteristics are summarized in Table 1. The median [IQR] for patient age was 4 years [2 months—10 years]. Just over half of the cannulated patients had a congenital heart defect (56.7%). All but two patients (93%) passed their low-flow and clamp trials and were subsequently decannulated, with 66% of all included patients surviving to hospital discharge. Affinity pumps were utilized the most at 27/31 (87%), and the remaining 4 (13%) were supported on Cardiohelp-based circuits. Peripheral cannulation accounted for 22/31 (71%) of ECMO runs, while the remainder were central cannulations. All centrally cannulated patients (9/31, 29%) had an open chest at the time of their low-flow and clamp trials. The median [IQR] for ECMO duration was 206 [116–268] hours, at flow rates of 0.9 [0.44–2.62] L/min, and a cardiac index (CI) of 1.81 [1.53–2.13] L/min/m^2^ (in the 48 h preceding a trial).

**Table 1:** Patient and ECMO run characteristics.

Patient & Circuit Characteristics Median [IQR]	ECMO Runs *n* = 31	Circuit Intervention or Stroke *n* = 6	No Adverse Event *n* = 25	*p*-value
Age, years	4 [0.1–10]	0.1 [0.1–4]	1.5 [0.1–10]	0.722
Weight, kg	16 [3.18–38.1]	3.18 [2.6–16]	10 [3.6–37]	0.456
BSA, m^2^	0.3 [0.23–1.23]	0.2 [0.19–0.68]	0.44 [0.23–1.22]	0.517
Cannulation Indication, *n* (%)				0.774
Congenital Heart Defect	17 (56%)	4	13	0.664
Pulmonary Hypertension	4 (13%)	1	3	
Arrhythmia	3 (9%)	0	3	
Septic Shock	3 (9%)	0	3	
Respiratory Failure	4 (13%)	1	3	
Cannulation Site, *n* (%)				0.043*
Central	9 (29%)	4	5	
Peripheral	22 (71%)	2	20	
Pump Type, *n* (%)				>0.99
Affinity	27 (87%)	5	22	
Cardiohelp	4 (13%)	1	3	
Survival to Decannulation, *n* (%)	30 (97%)	6	24	>0.99
Survival to Discharge, *n* (%)	21 (66.7%)	4	17	>0.99
ECMO characteristics				
Total Duration, hours	206 [116–268]	203 [115–209]	245 [108–325]	0.318
Duration Prior to Trial, hours	156 [100–210]	153 [74–201]	182 [98–251]	0.314
Flow Rate, L/min**	0.9 [0.44–2.62]	0.44 [0.40–0.97]	0.75 [0.43–2.46]	0.398
Cardiac index, L/min/m^2^**	1.81 [1.53–2.13]	1.70 [1.34–2.18]	1.82 [1.53–2.16]	0.581

* *p* *<* *0.05* by Fisher Exact or Mann Whitney U test.

** Highest flow rate in the 48 h period before a trial.

IQR, interquartile range; ECMO, extracorporeal membrane oxygenation.

### 3.2 Trial characteristics

During these 31 ECMO runs, 65 trials were performed: 27 low-flow and 38 clamp trials. All low-flow trials were immediately followed by a clamp trial. Of all clamp trials, 26/38 (68%) were followed by a decannulation immediately after. This accounted for some patients undergoing more than one clamp trial, and one patient failing and passing away. The median ECMO duration prior to a low flow trial was 156 [100–210] hours, with the trials lasting 60 [60–180] mins and with flows of 0.2 [0.13–0.5] L/min, corresponding to a cardiac index of 0.63 [0.43–0.84] L/min/m^2^. Clamp trial durations were also similar, but less variable lasting 50 [30–60] mins. Clamp trial-to-decannulation times were 20 [3.25–73.25] hours. Table 2 shows the low-flow and clamp trial characteristics.

**Table 2:** Low-flow and clamp trial characteristics.

Variable Median [IQR]	Total Trials *n* = 65	Circuit Intervention or Stroke *n* = 11	No Adverse Event *n* = 54	*p*-value*
Low-flow trial duration, minutes	60 [60–180]	60 [60–180]	60 [44–180]	0.8369
Low-flow trial flow rate, L/min	0.2 [0.13–0.5]	0.22 [0.20–0.40]	0.20 [0.11–0.48]	0.7745
Clamp trial duration, minutes	58 [ 30–60]	60 [48–60]	48 [30–60]	0.3866
Number of heparin boluses	1 [1–2]	1 [1–2]	1 [1–2]	>0.9
Heparin bolus dose, units/kg	25 [15–50]	50 [15–50]	25 [15–50]	0.5622
Bivalirudin rate (pre-trial), mg/kg/hr	0.2 [0.12–0.28]	0.28 [0.15–0.28]	0.19 [0.11–0.23]	0.0793
Bivalirudin rate (post-trial), mg/kg/hr	0.2 [0.15–0.28]	0.28 [0.21–0.30]	0.19 [0.14–0.25]	0.1555

* *p* *<* *0.05* by Mann Whitney U test.

IQR, interquartile range.

### 3.3 Blood product administration

**Figure 2:**
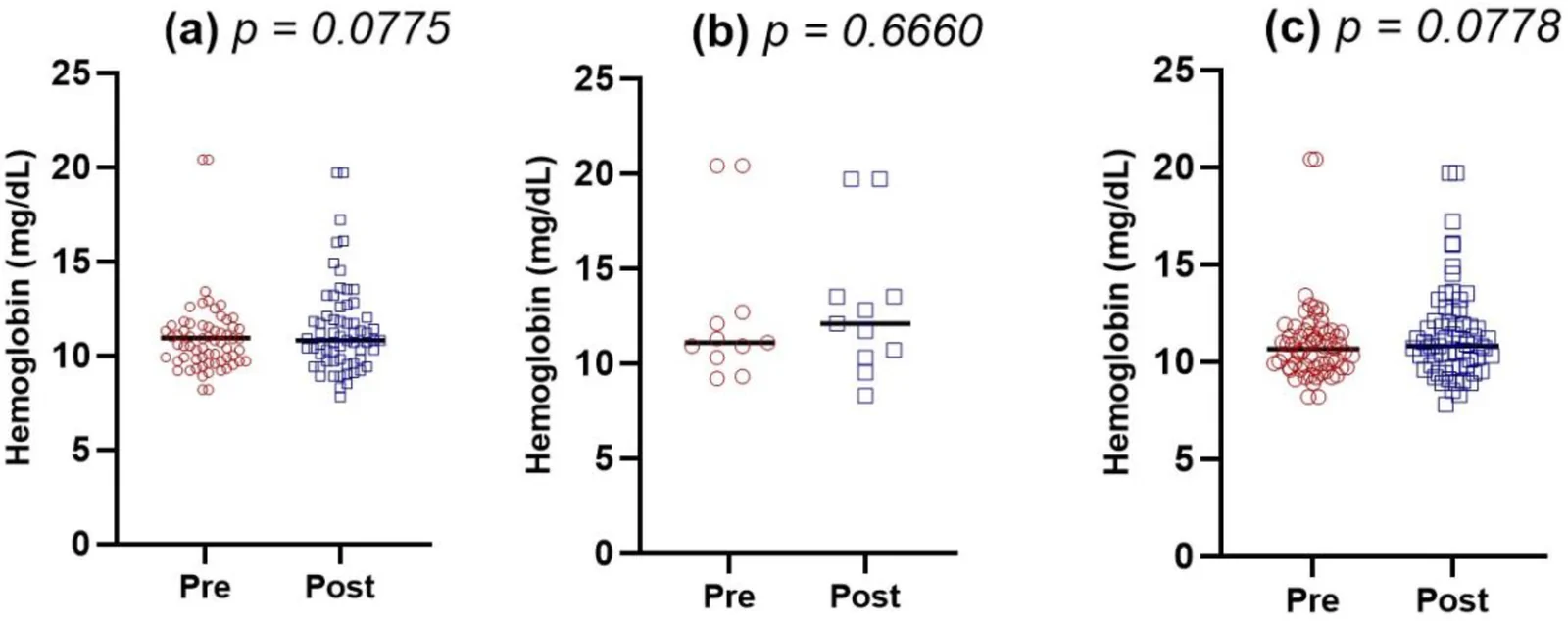
Comparison of hemoglobin levels pre- and post-trial. **(a)** Wilcoxon signed-rank test comparing hemoglobin levels pre and post trials in all studied ECMO runs, showing no significant difference, *p* = 0.0775. **(b)** Wilcoxon signed-rank test comparing hemoglobin levels pre and post trials in ECMO runs associated with post-trial circuit interventions or stroke, showing no significant difference, *p* = 0.666. **(c)** Wilcoxon signed-rank test comparing hemoglobin levels pre and post trials in ECMO runs not associated with post-trial circuit interventions or stroke, showing no significant difference, *p* = 0.0778.

Packed red blood cells (pRBCs) made up 80% of total blood product volume. In a paired analysis of the median [IQR] total volume of blood products transfused, a larger amount was administered following a trial [14.3 (15.1–24.5) mL/kg] vs. prior to a trial [5 (7.52–14.6) mL/kg] (*p* *<* *0.001*). These time periods were defined as the 24 h period pre-trial, and the 24 h period post-trial. The same analysis was performed using hemoglobin levels before and after every trial, which did not demonstrate a statistically significant difference, with pre [10.65 (9.7–11.5) mg/dL] vs. post [10.80 (9.7–12.1) mg/dL] (*p* *=* 0.0775) (Figure 2).

**Figure 3:**
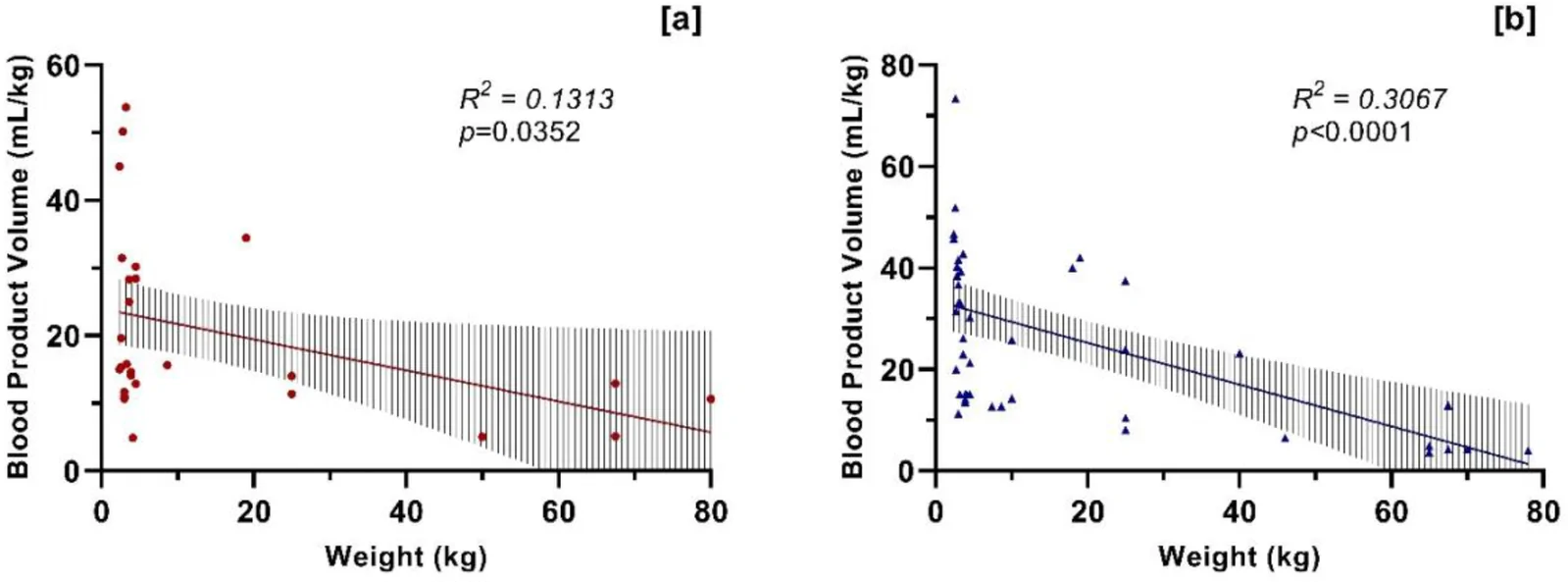
Association analysis of blood product volume administration and weight pre and post trials. **(a)** Linear regression analysis of pre-trial blood product volume administration and weight showed a significant association with lower weight, *p* = 0.0352 and **(b)** Linear regression analysis of post-trial blood product volume administration and weight showed a stronger significant association with lower weight, *p* < 0.0001.

Simple linear regression demonstrated a statistically significant correlation between patient weight and blood product administration. Thirty-eight trials were done in patients who weighed <15 kg, while the remaining 27 trials were in patients ≥15 kg. This analysis revealed that those <15 kg received more blood product volumes, which held true pre-trial with a correlation coefficient R^2^ [CI] of 0.13 [−0.44, −0.02] *(p* *=* 0.0352*)* **(**Figure 3 A), as well as post-trial 0.31 [−0.59, −0.23] *(p* *<* 0.001*)* **(**Figure 3 B). Although the same analysis done post-trial demonstrated higher median [IQR] hemoglobin levels for the lower weight group (11.2 [10–13.5] vs. 10.7 [9.6–11.3] mg/dL, *p* *=* 0.077), it was not statistically significant.

While there was no difference in the blood products received prior to a trial between peripheral and centrally cannulated patients, the latter received a significantly higher blood product volume post-trial (10.4 [0–22.2] vs. 38.9 [20.8–41.3] mL/kg, *p* *<* 0.0001). When performing a paired comparison of pre- and post-trial blood product administration by cannulation modality, both the peripherally and centrally cannulated patients received more blood post-trial, consistent with the previous analysis done; median difference [CI] [3.168 (0.48–6.79) mL/kg, *p* *=* 0.033] and [20.38 (10.88–29.89) mL/kg, *p* *<* 0.001], respectively.

We compared trials where average ECMO flows were <1L/min (39/65 trials), and ≥1 L/min (26/65 trials). In the pre-trial period, those flowing <1L/min received more blood products with a median [IQR] 14.8 [0–28.3] mL/kg than the ≥1 L/min at 0 [0–5] mL/kg (*p* *<* 0.0001). The same was true in the post-trial period, with the lower flow group receiving 24.4 [14.0–39.6] mL/kg than the higher flowing group at 4.2 [0–12.9] mL/kg (*p* *=* 0.0001). However, when analyzing the <1 L/min flow group independently, more blood was given post-trial at 24.4 [14.0–39.6] mL/kg than pre-trial at 14.8 [0–28.3] mL/kg (*p* *=* 0.001). The same is true for the ≥1 L/min group (*p* *=* 0.0165). The latter analysis is consistent with the previously established higher volume of blood product administration given post-trial across the board. Linear regression analysis with R^2^ [CI] results between trial flow rate and blood volume administration both pre- [R^2^ 0.08 (−3.86, 25.24), *p* *=* 0.143] and post-trial [R^2^ 0.06 (−8.34, 35.84), *p* *=* 0.211] was without significant correlation. Lastly, there was no significant correlation between trial duration, and the volume of blood products administered pre-trial [R^2^ 0 (−0.12, 0.22), *p* *=* 0.557], and post-trial [R^2^ 0 (−0.20, 0.21) *p* *=* 0.973].

### 3.4 Anticoagulation

Bivalirudin infusion rate 24 h pre-trial median [IQR] was 0.2 [0.12–0.28] mg/kg/hr and 0.22 [0.15–0.28] mg/kg/hr post-trial (*p* *=* 0.705). The median [IQR] UFH bolus dose was 25 [15–50] units/kg (Table 2). The PTT values pre- and post-trial were 64 [54–69] seconds, and 66 [57–76] seconds, respectively (*p* *=* 0.1038). TEG R-times pre- and post-trial were 9 [7.1–10] seconds, and 9.1 [7.5–9.9] seconds, respectively (*p* *=* 0.808).

**Figure 4:**
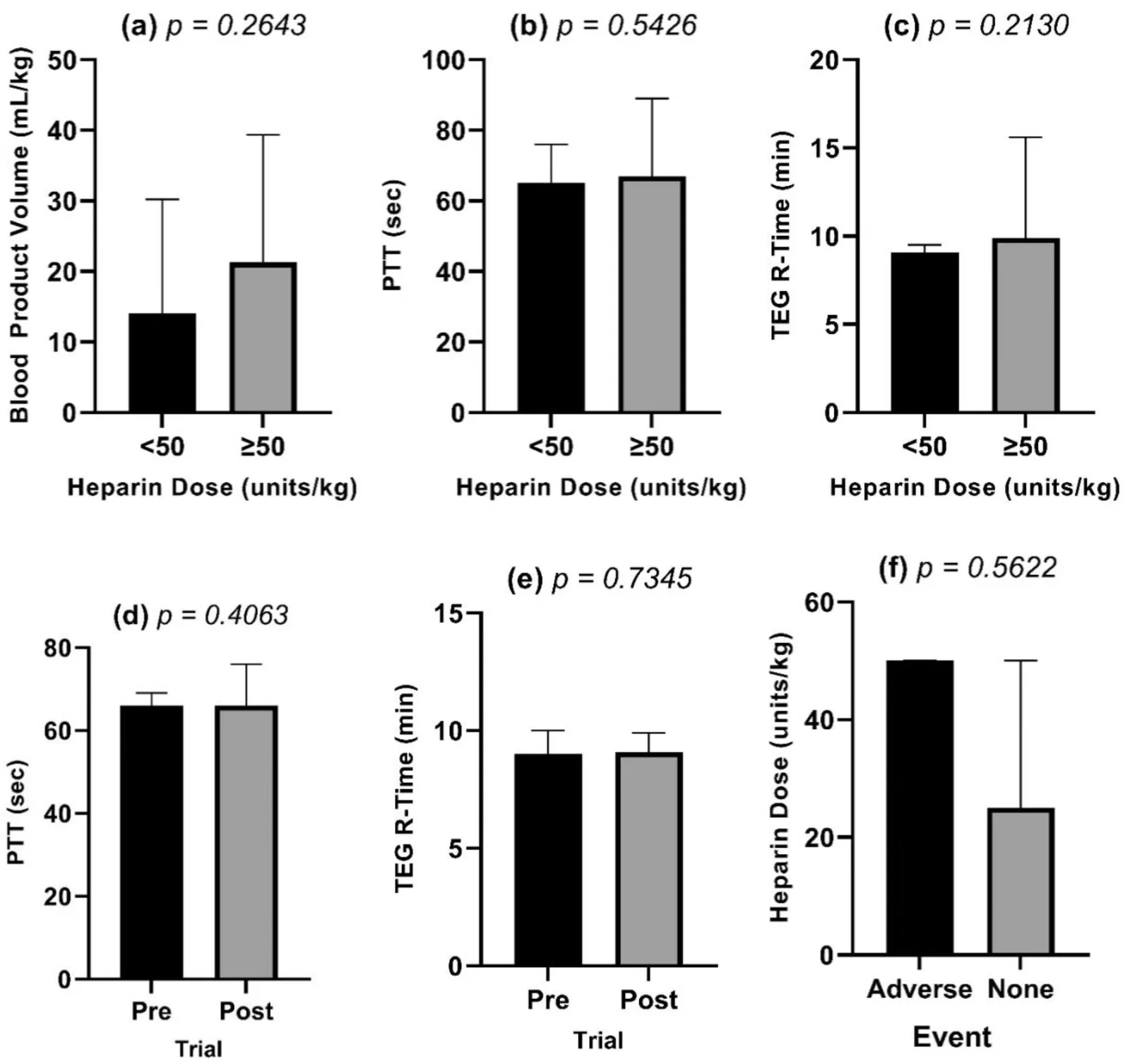
Comparison of laboratory markers pre and post trials and by heparin bolus dose. **(a)** Mann–Whitney *U*-test comparing blood product volume administered pre and post trials, showing no difference by high dose (≥50 units/kg) vs. low dose (<50 units/kg) heparin boluses, *p* = 0.2643. **(b)** Mann–Whitney *U*-test comparing post-trial aPTT (activated partial thromboplastin time) levels, showing no difference by high dose (≥50 units/kg) vs. low dose (<50 units/kg) heparin boluses, *p* = 0.5426. **(c)** Mann–Whitney *U*-test comparing post-trial TEG R-time (thromboelastography reaction time), showing no difference by high dose (≥50 units/kg) vs. low dose (<50 units/kg) heparin boluses, *p* = 0.2130. **(d)** Mann–Whitney *U*-test comparing PTT levels pre and post trials showing no significant difference, *p* = 0.4063. **(e)** Mann–Whitney *U*-test comparing TEG R-time pre- and post-trials showing no significant difference, *p* = 0.7345. **(f)** Mann–Whitney *U*-test comparing heparin bolus doses between runs associated with post-trial adverse events, circuit intervention or stroke, and those without showing no significant difference, *p* = 0.5622.

Overall, there was no significant difference in the median [IQR] PTT [66 (53–71) seconds, *p* *=* 0.406] or the TEG R-time [9 (7–9) minutes, *p* *=* 0.735] values before and after each trial, and both values remained within our institutional therapeutic targets before and after low-flow and clamp trials. Looking further at the correlation between anticoagulation dosing and laboratory monitoring, there was no difference in PTT or TEG R-time between heparin boluses <50 units/kg and ≥ 50 units/kg. Heparin dose was not associated with a difference in the volume of blood product administration. Similarly, there was no difference in either of these values in the 24 h before and after every trial (Figure 4). There was also no difference in the median [IQR] heparin dose given during trials between centrally [25 (13.75–50) units/kg] and peripherally [50 (25–50) units/kg] cannulated patients (*p* *=* 0.108).

Bivalirudin infusion rate median [IQR] pre- [0.2 (0.12–0.18) mg/kg/hr] and post-trial [0.21 (0.16–0.28) mg/kg/hr] were not different (*p* *=* 0.525). Linear regression analysis of PTT and TEG-R time with bivalirudin infusion rates demonstrated values within the therapeutic range before and after trials. Pre-trial bivalirudin infusion was higher in the centrally cannulated patients at 0.28 [0.15–0.28] mg/kg/hr compared to the peripherally cannulated at 0.19 [0.10–0.22] mg/kg/hr (*p* *=* 0.043). However, the post-trial bivalirudin infusion rates were not different in central [0.23 (0.17–0.30) mg/kg/hr] vs. peripheral cannulation (0.2 [0.11–0.25] mg/kg/hr, *p* *=* 0.341). Linear regression analysis between bivalirudin infusion rates and ECMO flow rates, low-flow trial rates as well as trial duration remained statistically unchanged before and after each trial. Additionally, on subgroup analysis by weight, there was not a statistically significant difference in either heparin bolus dose, or bivalirudin infusion rates pre- and post-trial between patients weighing <15 kg or ≥15 kg. There was no association between blood product volume administration and low flow trial flow rates pre-trial, post-trial, bivalirudin infusion rates, heparin bolus dose, PTT and TEG R-times.

### 3.5 Circuit interventions and patient stroke

Eight (12%) of the 65 trials experienced thrombosis requiring circuit interventions, which included 6 cannula/connector changes, and 2 pump exchanges, between 4 different patients. All were performed within 24 h following a low-flow trial, and none were on the same circuit. In all eight trials, the median [IQR] time to circuit intervention was 1.5 [0.75–3.5] hours following the trial. In terms of patient stroke incidence as identified on post-trial head imaging, a total of 3 patients experienced a significant stroke event, all were ischemic in nature. There was no overlap between patients who had a circuit intervention and those who experienced a stroke event. When comparing bivalirudin infusion rates, heparin bolus dose and frequency between patients who did and did not have a stroke or circuit intervention, no statistically significant difference was found (Table 2).

There was no difference observed in the blood volume administered pre- and post-trial between those where either a circuit intervention occurred, or a stroke was identified. However, when performing a paired comparison between the pre- and post-trial blood volume within each cohort, the trials associated with either a circuit intervention or stroke (median difference 25.67 mL/kg, CI [12.40–37.97], *p* *=* 0.004) and those without (0.33 mL/kg, [1.79–9.08], *p* = 0.007), both had higher transfusion volumes in the post-trial periods.

There was no difference between the trials associated with either a circuit intervention or a stroke and those without in terms of the median [IQR] heparin boluses administered (50 [15–50] units/kg vs. 25 [15–50] units/kg, *p* *=* 0.562), or the bivalirudin infusion rates pre-trial (0.28 [0.15–0.28] mg/kg/hr vs. 0.2 [0.11–0.23] mg/kg/hr, *p* *=* 0.078) and post-trial (0.28 [0.21–0.3] mg/kg/hr vs. 0.19 [0.14–0.25] mg/kg/hr, *p* *=* 0.156). Regarding the laboratory values, there was no difference in the PTT values pre- and post-trial, or hemoglobin values. For the TEG R-time, the group with either a circuit intervention or stroke had a shorter median [IQR] 7 [6–7.8] mins, than the group without any at 8.5 [7.2–10.9] mins (*p* *<* 0.05). There was no such difference post-trial. ECMO flow rate, trial duration, and trial flow rates were also not statistically significant between the two groups. It is notable, however, that 8/11 (72%) patients who had a circuit intervention or stroke were centrally cannulated, and were also <15 kg, both which were previously identified as being associated with higher blood product volume administration.

## 4 Discussion

Despite the advantages of bivalirudin over UFH, concerns over efficacy during low-flow states due to its enzymatic proteolysis, continue to limit its widespread adoption (9, 10). Some centers that utilize bivalirudin for patients who are sub-therapeutic on UFH, will switch back to UFH during these trials. To date, there remains a paucity of evidence pertaining to anticoagulation management for patients on bivalirudin during decannulation readiness trials. In this paper, we analyzed our institutional practice of administering additional UFH boluses during low flow and clamp trials. We observed an incidence of circuit interventions, adverse neurological events, and bleeding that is similar to that currently reported (12, 13). Our results demonstrate that our approach to anticoagulation with the addition of UFH boluses during these trials appears to be safe. In our analysis, we identified an association between lower patient weight, and an increased blood product administration, however there was no identifiable bleeding risk, stroke risk, or increased incidence of circuit thrombosis.

One universal finding was the larger volume of blood administered post-trial in comparison to prior. Given the paucity of data in the medical records as to the indication for these transfusions, we further analyzed the change in hemoglobin levels in the 24 h before and after each trial, for which there was no significant difference. Although blood product administration increased following trials, there was no corresponding decrease in hemoglobin concentration. This suggests that the increased transfusion volume likely reflects institutional transfusion practices surrounding ECMO decannulation and optimization of oxygen-carrying capacity, particularly in smaller postoperative patients, rather than clinically significant bleeding attributable to the anticoagulation strategy. Increasing hemoglobin content subsequently increased oxygen delivery, thereby increasing the threshold for development of metabolic acidosis as ECMO support decreased. The smaller weight patient group had higher transfusion volumes post trials as compared to the larger patients. These differences persisted even after controlling for the pre-trial transfusion volumes. This is likely due to the increased risk of neonates and infants developing iatrogenic anemia from blood draws (13), surgical site blood loss, and are more likely to benefit from transfusions that optimize their ability to successfully separate from ECMO support.

As for cannulation modality, centrally cannulated patients had higher transfusion volumes compared to those peripherally cannulated. All of our centrally cannulated patients also had an open chest. Approximately half of the lower-weight patients were centrally cannulated, which likely contributed to higher transfusion requirements independent of bleeding. These findings are consistent with a recent analysis by Ankola et al. (14), describing an increased risk for bleeding events in centrally cannulated patients. That study did not find an association between bleeding and heparin dosing. Within our own data, we first compared whether centrally cannulated patients received different anticoagulation dosages to those peripherally cannulated. We found no difference in the UFH bolus dose or frequency between both groups. Bivalirudin dosing ranges were similar. There was no difference in their hemoglobin levels, heparin and bivalirudin dosing. It is difficult to ascertain how much of the higher blood product administration in central cannulation was secondary to those patients having open chests, lower body weight, or other potential causes. Our study was not powered to separate these differences, nor to perform a separate analysis on the CHD patients.

There is growing experience with bivalirudin as the primary anticoagulant for pediatric ECMO, with no difference in bleeding or thromboembolic events (15, 16), and perhaps even less bleeding events when compared to UFH (17). Nevertheless, some patients have been reported to develop intracardiac thrombi while on bivalirudin, due to stagnation of blood flow in chambers that may be inadequately decompressed, however none were found in our cohort (18). We assessed whether our approach to anticoagulation was clinically within the therapeutic window, by measuring the incidence of circuit thrombosis and stroke. We observed an incidence of post-trial circuit intervention in a fourth of the studied cohort. Although we did not have a comparison group, this proportion is lower than that described in our prior institutional experience (4). This rate of circuit intervention is also similar to that reported by Dalton et. al (13) in the multi-center prospective cohort study evaluating factors associated with bleeding and thrombosis in pediatric ECMO. Stroke occurred in less than a tenth of our cohort, all of which were ischemic. Because neuroimaging was performed only when clinically indicated rather than routinely after trials, the temporal relationship between neurologic events and the trials could not be established, and clinically silent neurologic events may have been missed. We anticipated that factors such as lower ECMO flow rates, and longer trial durations, were likely to increase risk for circuit thrombosis, but did not observe such associations in our analyses. This is also in the setting of relatively uniform bivalirudin infusion rates and UFH boluses across all trials. There was no difference in the bivalirudin infusion rate or UFH dose given in patients experiencing circuit thrombosis or stroke, when compared to those without either adverse event. Immediate post-UFH coagulation studies were not routinely obtained because laboratory turnaround times exceeded the interval between repeat UFH boluses during prolonged trials. Consequently, anticoagulation was assessed using post-trial laboratory values rather than immediate post-bolus measurements. There were also no differences in the hemoglobin values or blood products volumes before and after a trial between this patient population, and those without any adverse events.

There are no established guidelines for bivalirudin dosing in ECMO, but some institutional protocols have been described (6, 19, 20). We measured PTT, and a comprehensive TEG profile (reaction-time, maximum amplitude, and G-value), demonstrated no statistically significant differences in the 24 h period before or after each trial. Our patients spent the majority of their ECMO run within the therapeutic range, suggesting that no sustained differences in overall anticoagulation were observed before and after the low-flow or clamp trials. Similar consistency in lab testing of bivalirudin vs. heparin was reported my Hamzah et al. (17) Additionally, despite the higher price of DTIs, such stability allowed for decreased overall cost due to less testing and antithrombin-III replacements.

Limitations of our study begin with its retrospective and non-randomized nature. Our linear regression was unadjusted due to sample size. We were unable to compare our results with those of a heparin-only control group. As with any retrospective observational study, residual confounding cannot be excluded. We were unable to account for several patient-, circuit-, and management-related factors that may have influenced bleeding, thrombosis, and circuit intervention, including individualized heparin dosing based on clinical judgment, peri-decannulation management, coagulation and inflammatory variables, clinically indicated rather than routine neuroimaging, subjective assessment of circuit thrombus burden, and the absence of immediate post-heparin laboratory measurements. Accordingly, the observed associations or lack of, should be interpreted as hypothesis-generating rather than causal. We were unable to account for all patient-specific factors influencing bleeding and thrombosis or the individualized clinical decision-making that informed heparin bolus dosing. Accordingly, residual confounding cannot be excluded. Retrospective quantification of clinically significant bleeding was limited by inconsistent documentation and the inability to distinguish expected postoperative chest tube output from pathologic bleeding. Therefore, transfusion requirements and changes in hemoglobin concentration were used as surrogate measures. Objective measures of baseline circuit thrombus burden and oxygenator performance were not collected and therefore could not be evaluated. We relied on a major thrombotic event that necessitated a circuit intervention as a primary outcome. Because circuit intervention was based on multidisciplinary clinical assessment rather than standardized objective criteria, variability in clinical decision-making may have influenced this outcome. Doing so may be an oversight, as it can be considered a crude estimate of circuit thrombosis and does not finely assess deposit and fibrin laydown after trials. Unfortunately, there is no current standard or objective guideline on tracking ECMO circuit depositions, an evaluation that continues to be subjective and variable. Detailed intra-trial hemodynamic variables were beyond the scope of this study, which focused on the safety of anticoagulation during low-flow and clamp trials rather than patient hemodynamic responses to the trials themselves. We attempted to directly compare the same outcomes between low-flow and clamp trials, as clamp trials are theoretically more likely to contribute to circuit thrombosis than low-flow ones. However, given that both were performed within a few hours of each other, we could not analyze them separately. Similarly, post-trial transfusion requirements likely reflect both the anticoagulation strategy and routine peri-decannulation management, limiting attribution of these outcomes to the UFH bolus alone. In summary, our above results reflect the combined effect of both types of trials. Our sample size also limited our ability to perform adequately powered subgroup analyses to evaluate whether prolonged ECMO support or prolonged low-flow/clamp trial durations were associated with adverse outcomes. It was also due to this limitation that we were unable to compare outcomes based on underlying patient diagnoses. A strength of this study is the consistency in performing each trial. Low-flow trials had significantly less variation when compared with patient flows during full support. This also applied to trial duration, heparin bolus administration, as well as time between heparin boluses in longer trials. We relied on and reported a comprehensive laboratory profile via both PTT and TEGs, to ensure monitoring consistency. Our overall ECMO patient survival rate was congruent with the currently reported body of evidence.

## 5 Conclusion

In our single-center experience, the addition of UFH boluses during low-flow and clamp trials in pediatric patients receiving bivalirudin was not associated with clinically significant changes in bleeding surrogates, circuit intervention, or stroke. These findings suggest that this anticoagulation strategy is feasible in this setting but should be interpreted in light of the study's retrospective design, small sample size, and potential for residual confounding. Larger prospective multicenter studies are needed to validate these findings and better define optimal anticoagulation strategies during low-flow and clamp trials.

## Data Availability

The raw data supporting the conclusions of this article will be made available by the authors, without undue reservation.

## References

[1] Neunert C Chitlur M Van Ommen CH. The changing landscape of anticoagulation in pediatric extracorporeal membrane oxygenation: use of the direct thrombin inhibitors. Front Med. (2022) 9:887199. 10.3389/fmed.2022.887199 PMC9299072 35872781

[2] Larabee SM Hollinger LE Vogel AM. Systemic anticoagulation in ECMO. Semin Pediatr Surg. (2023) 32(4):151333. 10.1016/j.sempedsurg.2023.151333 37967498

[3] Ozment CP Scott BL Bembea MM Spinella PC. Anticoagulation and transfusion management during neonatal and pediatric extracorporeal membrane oxygenation: a survey of medical directors in the United States. Pediatr Crit Care Med. (2021) 22(6):530–41. 10.1097/PCC.0000000000002696 33750092

[4] Schill MR Douds MT Burns EL Lahart MA Said AS Abarbanell AM. Is anticoagulation with bivalirudin comparable to heparin for pediatric extracorporeal life support? Results from a high-volume center. Artif Organs. (2021) 45(1):15–21. 10.1111/aor.13758 32557733

[5] Bembea MM Annich G Rycus P Oldenburg G Berkowitz I Pronovost P. Variability in anticoagulation management of patients on extracorporeal membrane oxygenation: an international survey. Pediatr Crit Care Med. (2013) 14(2):e77–84. 10.1097/PCC.0b013e31827127e4 23287906 PMC3567253

[6] Rabinowitz EJ Ouyang A Armstrong DR Wallendorf M Said AS. Poor reliability of common measures of anticoagulation in pediatric extracorporeal membrane oxygenation. Asaio j. (2022) 68(6):850–8. 10.1097/MAT.0000000000001582 34581287

[7] Wieruszewski PM Brinkman HM Ortoleva JP Ripoll JG Pieterick SE Damico KE. Dosing reliability of direct thrombin inhibitors in extracorporeal membrane oxygenation: a systematic review and meta-analysis. ASAIO J. (2025) 71(9):691–700. 10.1097/MAT.0000000000002512 40673548

[8] Robson R White H Aylward P Frampton C. Bivalirudin pharmacokinetics and pharmacodynamics: effect of renal function, dose, and gender. Clin Pharmacol Ther. (2002) 71(6):433–9. 10.1067/mcp.2002.124522 12087346

[9] Warkentin TE Greinacher A. Heparin-induced thrombocytopenia and cardiac surgery. Ann Thorac Surg. (2003) 76(2):638–48. 10.1016/S0003-4975(03)00756-2 12902132

[10] Warkentin TE Greinacher A Koster A. Bivalirudin. Thromb Haemost. (2008) 99(5):830–9. 10.1160/TH07-10-0644 18449412

[11] Lin JC Barron LM Vogel AM Colvin RM Baltagi SA Doctor A. Context-Responsive anticoagulation reduces complications in pediatric extracorporeal membrane oxygenation. Front Cardiovasc Med. (2021) 8:637106. 10.3389/fcvm.2021.637106 34179125 PMC8224528

[12] Friedman ML Bell MJ Brooks BA Himebauch AS Kamerkar A LaRovere K. Plasma biomarkers of brain injury in critically ill children receiving extracorporeal membrane oxygenation. JAMA Pediatr. (2026) 180(6):639–49. 10.1001/jamapediatrics.2026.0015 41770542 PMC12954597

[13] Dalton HJ Reeder R Garcia-Filion P Holubkov R Berg RA Zuppa A. Factors associated with bleeding and thrombosis in children receiving extracorporeal membrane oxygenation. Am J Respir Crit Care Med. (2017) 196(6):762–71. 10.1164/rccm.201609-1945OC 28328243 PMC5620676

[14] Ankola AA Bailly DK Reeder RW Cashen K Dalton HJ Dolgner SJ. Risk factors associated with bleeding in children with cardiac disease receiving extracorporeal membrane oxygenation: a multi-center data linkage analysis. Front Cardiovasc Med. (2022) 8:812881. 10.3389/fcvm.2021.812881 35097029 PMC8792849

[15] Nagle EL Dager WE Duby JJ Roberts AJ Kenny LE Murthy MS. Bivalirudin in pediatric patients maintained on extracorporeal life support. Pediatr Crit Care Med. (2013) 14(4):e182–8. 10.1097/PCC.0b013e31827200b6 23648880

[16] Ranucci M Ballotta A Kandil H Isgrò G Carlucci C Baryshnikova E. Bivalirudin-based versus conventional heparin anticoagulation for postcardiotomy extracorporeal membrane oxygenation. Crit Care. (2011) 15(6):R275. 10.1186/cc10556 22099212 PMC3388709

[17] Hamzah M Jarden AM Ezetendu C Stewart R. Evaluation of bivalirudin as an alternative to heparin for systemic anticoagulation in pediatric extracorporeal membrane oxygenation. Pediatr Crit Care Med. (2020) 21(9):827–34. 10.1097/PCC.0000000000002384 32404633

[18] Ranucci M. Bivalirudin and post-cardiotomy ECMO: a word of caution. Crit Care. (2012) 16(3):427. 10.1186/cc11314 22574927 PMC3580606

[19] Snyder CW Goldenberg NA Nguyen ATH Smithers CJ Kays DW. A perioperative bivalirudin anticoagulation protocol for neonates with congenital diaphragmatic hernia on extracorporeal membrane oxygenation. Thromb Res. (2020) 193:198–203. 10.1016/j.thromres.2020.07.043 32763642

[20] Chomat MR Swanson K Barton K Douds M Said AS. Management of bivalirudin dosing and replacement fluid during therapeutic plasma exchange in children on extracorporeal membrane oxygenation. ASAIO J. (2024) 70(3):e31–e7. 10.1097/MAT.0000000000002094 38029748

